# Combination therapies in Myeloproliferative Neoplasms: why do we need them and how to identify potential winners?

**DOI:** 10.1111/jcmm.12202

**Published:** 2013-12-23

**Authors:** Donal McLornan, Claire Harrison

**Affiliations:** Guy's and St Thomas’ NHS Foundation Trust Guy's Hospital, Great Maze PondLondon, UK

## Abstract

The myeloproliferative neoplasms (MPN) are clonal myeloid disorders characterized by proliferation of mature myeloid cells, such that in polycythaemia vera (PV), the red cell proliferation dominates, platelets in essential thrombocythaemia (ET) and in myelofibrosis (MF), there may be cytopenia or proliferation, but the characteristic feature is the strikingly abnormal bone marrow stroma. These entities have a tendency to show phenotypic mimicry and may transform from one to another, for example, 20–30% of patients with PV are likely to develop MF. The significant event in this field was the recognition that Janus Kinase-2 (JAK2) activation was highly prevalent, followed by the description of the *JAK2V617F* mutation in 2005 (*vide infra),* which stimulated renewed interest in disease biology. Janus Kinase-2-targeted therapies have led to marked improvements for patients with this condition. However, it is obvious that the pathogenesis of these complex disorders reaches beyond this mutation; only 50–60% of patients with ET, for example, have the *JAK2* mutation and several additional mutations have been described, which are of relevance in both the pathogenesis and clinical phenotype of these conditions.

The therapeutic benefits seen with the effect of JAK2 inhibitors are striking, including reduction of massive splenomegaly, resolution of constitutional symptoms and prolongation of survival as seen with Ruxolitinib, the first of the class JAK1/2 inhibitors 1–2. Such improvements, however, are not of the same magnitude as the magnitude of benefits associated with BCR/ABL inhibition in chronic myeloid leukaemia, for example. Most likely, this reflects a number of issues; firstly, that none of the inhibitors yet developed is specific for mutant JAK2, and secondly, that JAK2 activation or its consequence is not the only pathogenic mechanism operating in these intriguing disorders. This has several important implications for this field: we need to better understand the disease biology and develop systems for testing other novel therapies, either alone or in combination. In this issue, two papers (Choong *et al*. and Bartalucci *et al*. 3–4) describe different approaches to the issue of testing and evaluating drug combinations in MPN and demonstrate benefits *in vitro*, which probably merit clinical investigation. What are the potential pathways of interest?

Constitutive activation of JAK2 in MPN drives multiple downstream pathways, including the signal transducer and activator of transcription (STAT) 3/STAT5, mitogen-activated protein kinases (MAPK), extracellular signal–regulated kinase (ERK) and Phosphatidylinositol 3-kinase (PI3)/Akt/Mammalian target of Rapamycin (mTOR) pathways 5–8. Augmented ‘genetic instability’ and epigenetic modification may ensue (as described in Fig. 1). This genetic instability may subsequently facilitate disease progression. Activation of the PI3K pathway leads to pleiotropic effects on cellular proliferation, metabolism, intracellular trafficking and survival 9–10. Recent work has demonstrated elevated levels of phospho-STAT5, phospho-Akt and phospho-mTOR in progenitor cells derived from MPN patients 11. Studies of both ‘knock-in’ *JAK2*V617F murine models and MPN patient samples have documented increased reactive oxygen species (ROS) generation secondary to Akt-mediated down-regulation of catalase activity, with an accompanying increase in double-stranded breaks (DSB) 12. Furthermore, the PI3K/Akt/mTOR pathway is an important mediator of cellular drug resistance in both solid and haematological malignancies 13,14. The PI3K/Akt/mTOR pathway is therefore an attractive therapeutic target in patients with MPN.

**Figure 1:**
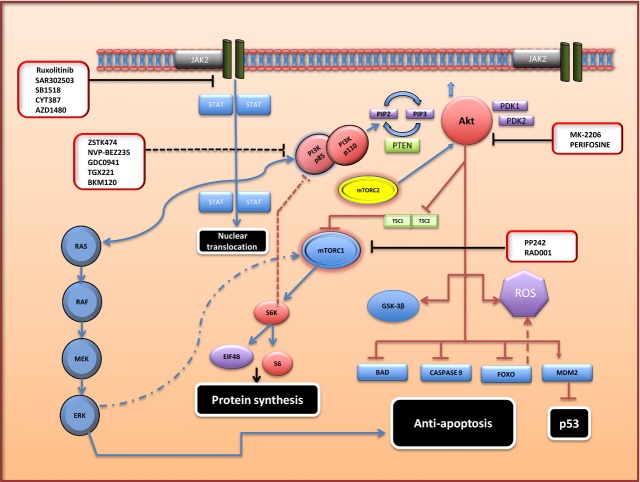
Potential Targets in the Myeloproliferative cell Janus Kinase-signal transducer and activator of transcription (JAK-STAT) and PI3K/Akt/Mammalian target of Rapamycin (mTOR) pathways. Constitutive activation of JAK2 in myeloproliferative neoplasms drives multiple downstream pathways, including the STAT3/STAT5, mitogen activated protein kinases (MAPK), extracellular signal–regulated kinase (ERK) and Phosphatidylinositol 3-kinase (PI3)/Akt/mTOR pathways. Multiple effects occur promoting cell growth, survival and metabolism. Potential therapeutic agents affecting various targets in these pathways are highlighted. *PIP2 -*phosphatidylinositol-4, 5-triphosphate; *PIP3 -*phosphatidylinositol-3, 4,5-triphosphate; *PTEN-* Phosphatase and tensin homologue; *mTORC* – mTOR complex*; PDK -*3′phosphoinositide-dependent kinase; *TSC*-Tuberose Sclerosis; *EIF4B*- Eukaryotic translation initiation factor 4B; *GSK3*β*-*Glycogen synthase kinase 3; *ROS* -Reactive Oxygen Species; *FOXO*- Forkhead Box Class O; *BAD*-Bcl-2-associated death promoter; *Mdm2-*Mouse double minute 2 homologue.

Three main classes of PI3Ks, class I, II and III, exist within cells. Class I PI3Ks, heterodimers consisting of a regulatory subunit (p85) and catalytic subunit (p110), are activated by cell surface receptors, including key receptor Tyrosine Kinases (RTKs) such as JAK, and are well characterized. Three isoforms of the p110 catalytic subunit exist: p110α, β and δ. Activation of class I PI3Ks induces phosphorylation of the 3′-hydroxyl group of the inositol ring of phosphatidylinositol-4, 5-triphosphate, PI 4–5 P2 (PIP2), leading to the generation of phosphatidylinositol-3, 4,5-triphosphate, PI 3,4 P3 (PIP3) 9–10. PIP3 promotes Akt translocation to the plasma membrane and subsequent phosphorylation of threonine 308 (Thr308) by 3′phosphoinositide-dependent kinase-1 (PDK1) occurs, leading to activation 9–18. In general, full activation of Akt requires not only phosphorylation of Thr308 but also Serine 473 (Ser473) within the hydrophobic C-terminal domain by PDK2 19. Akt activation inhibits Tuberose Sclerosis (TSC) 1/TSC2 complex function and leads to enhanced mTOR activity. Moreover, diverse effects outside the PI3K/Akt/mTOR ‘vertical pathway’ can also occur following Akt activation, predominantly driving a proliferative and anti-apoptotic state. These include inhibition of both procaspase-9 and BAD, a pro-apoptotic BCL-2 family member. In addition, inhibition of Forkhead Box Class O (FOXO) transcription factors and nuclear translocation of Mdm2, with resultant down-regulation of p53 transcriptional activity, may follow 9–22. In the context of MPN, It is well documented that constitutive Akt activation exists in both murine transgenic and ‘knock in’ *JAK2* V617F models and in MPN patient samples 23.

Downstream, mTOR functions as a key regulatory serine/threonine kinase that also modulates cellular proliferation, metabolism and apoptotic threshold. Two distinct cellular complexes exist, mTORc1 (comprising mTOR, Raptor, MLST8, DEPTOR and PRAS40) and mTORc2 (comprising mTOR, RICTOR, GβL and mSIN1), which possess differential sensitivities to the ‘first-generation’ mTOR inhibitor rapamycin 24. mTOR is subject to regulation by both ‘Akt-dependent’ and ‘Akt-independent’ mechanisms, for example, in addition to PI3K/Akt-induced mTORc activation, the MEK-ERK signalling pathway may also stimulate mTORc1 activity 25–26. In general, following activation, mTORc1 stimulates two key effector ribosomal S6 Kinases, S6K1 and S6K2. Substrates of S6K1 include the ribosomal protein, S6, and eukaryotic initiation factor 4B (EIF4B) and pharmacological inhibition can thus impair cap-dependent mRNA translation and induce cell cycle arrest, hence making mTORC1 an attractive therapeutic target. mTORC2 possesses PDK2 activity and can phosphorylate Akt Ser473 and influence Akt function 10,26.

Multiple mTOR inhibitors have entered the clinical arena across a spectrum of malignancies, affording variable therapeutic efficacy 28,29. Existence of alternative Akt/mTOR-associated regulatory pathways and aberrant PI3K feedback may lead to suboptimal anti-tumoral effects with single-agent mTOR inhibitors. Results from a phase 1/2 trial of the allosteric mTOR inhibitor everolimus in 30/39 evaluable patients with MF demonstrated modest clinical activity as regards reductions in splenic dimensions and amelioration of constitutional symptoms, dependent upon the objective disease response criteria utilized 31. No reduction in *JAK2*V617F allele burden was detected. Subsequent *in vitro* work explored the effects of both everolimus and the ATP-competitive mTOR inhibitor PP242 alone or in combination with JAK inhibitors (JAKi) in both murine and human *JAK2*V617F-mutated cell lines and primary MPN progenitor cells. Inhibition reduced both proliferation and colony formation ability of *JAK2*V617F mutated cell lines and patient-derived progenitor cells in an apparent dose-dependent manner. Effects were mainly cytostatic, although PP24 at higher dosages induced apoptosis. In addition, exploratory combination experiments with JAKi demonstrated synergistic activity in *JAK2*V617F-mutated cell lines and enhanced inhibition of EEC formation.

The two following papers concerning potential usage of targeted agents against the P13/Akt/mTOR axis in MPN, both alone and in combination with JAKi therapy, provide further supportive evidence for this approach. In brief, Choong *et al*. performed a rigorous cellular screen assay utilizing the JAK inhibitors Ruxolitinib or TG101348 in combination with a broad panel of kinase inhibitors, to identify potential synergism 3. Both JAKi compounds displayed marked anti-proliferative synergy with the pan-class PI3K inhibitor (PI3Ki) ZSTK454 in a set of transformed *TPO*/*JAK2*-mutated BA/F3 cell lines, as determined by standard combination index (CI) calculations. Enhanced synergistic activity between JAKi and PI3Ki occurred when the concentration ratio of the two drugs favoured JAKi over PI3Ki, suggesting that inhibition of JAK2 signalling was essential for facilitating increased sensitization of cells to the PI3Ki. Significant synergistic results were also obtained with three alternative PI3Ki, NVP-BEZ235, GDC0941 and TGX221, but not with PI3Ki displaying specificity for γ- or δ-PI3K. Of note, in the absence of JAK inhibition, the transduced Ba/F3 cell lines were not particularly sensitive to PI3K inhibitors alone. In combination, JAKi and the PI3KI blocked both STAT3 and STAT5 signalling and key downstream PI3K targets p70 S6 kinase and s6 ribosomal protein. Of particular note, the synergistic activity between JAKI and PI3KI was observed in cells cultured in serum-free media, suggesting that PI13K activation is largely secondary to constitutive JAK2 activation. Murine MPN models containing Ba/F3 TpoR *JAK2*V617F cells were then used to elucidate the effects of each drug treatment, alone and in combination. Delayed onset of splenomegaly and enhanced survival were associated with combination JAK and PI3K inhibition. Of note, none of the MAP kinase inhibitors displayed significant synergism with the JAK2 inhibitors in this screening assay, emphasizing that the specific pathway targeted in drug combination studies with JAKi needs to be carefully elucidated.

Parallel *in vitro* characterization of combination treatment with a dual PI3K/mTOR inhibitor, BEZ235, and Ruxolitinib has been performed by Bartalucci *et al*. 4 BEZ235 is an imidazoquinoline derivative that inhibits both PI3K and mTOR by binding to the ATP-binding cleft of these enzyme complexes. Recently published work has demonstrated activity of BEZ235 against *BCR-ABL*-mutated cell lines and synergy with nilotinib *in vitro*, in addition to pro-apoptotic effects in both primary AML cells and AML cell lines 32–33. In this paper, murine cell line work demonstrated enhanced anti-proliferative effects of BEZ235 against Ba/F3 *JAK2*V617F and Ba/F3-EPOR-*JAK2*V617F cells compared with wild-type (WT) cells, with growth arrest in the GO/G1 phase. Higher dose concentrations were found to promote apoptosis in both the Ba/F3-EPOR *JAK2*V617F and human SET2 cell line. Progenitor cell clonogenic assays from *JAK2*V617F ‘knock-in’ mice bone marrow displayed enhanced sensitivity to the inhibitory effects of BEZ235 compared with the assays performed from bone marrow of wild-type mice, in particular the megakaryocytic progenitor colonies. Attenuation of phospho-mTOR expression was more evident in the BA/F3 EPOR-*JAK2*V617F cells compared with WT cells, although phosphorylation of 4EPB1 was reduced to a similar extent in both V617F and WT cells, indicating a direct effect of BEZ235. Akin to the work by Choong *et al*., CI studies determined synergistic activity between Ruxolitinib and BEZ235 in *JAK2*V617F mutated cell lines and a small number of PMF patient samples. Of particular note, phospho-protein western blot analysis of treated *JAK2*V617F cells revealed that this drug combination was associated with greater reductions in phosphorylated mTOR, 4EBP1 and STAT5 compared with each drug alone. Finally the group used novel MPN murine models to display enhanced survival, rapid reductions in splenomegaly and a decrease in the characteristic histopathological features of extramedullary haematopoiesis with dual PI3Ki/JAKi therapy.

Both papers provide evidence promoting the potential benefits of dual targeting of both the JAK and PI3/Akt/mTOR pathways in an attempt to abrogate maintenance of the MPN phenotype. Combining JAKi with PI3/Akt/mTOR inhibitors could not only improve clinical efficacy but may also lead to deeper ‘molecular’ responses within the MPN clonal population. Of particular note, the drug concentrations used in combination studies of Ruxolitinib and BEZ235 by the Bartalucci group were lower than the efficacious single-agent doses. This dose reduction may therefore translate into reduced toxicity affecting normal haematopoiesis, which may be a dose-limiting factor for patients on JAKi therapy. As described above, enhanced ROS generation may exist in MPN patients, in part, mediated by Akt activity. Recently published work has shown increased ROS generation and p47phox phosphorylation in neutrophils from *JAK2*V617F patients 34. This may contribute to both endothelial damage and MPN-associated thrombotic complications. Increased ROS generation also plays a significant role in MPN genetic instability and dual inhibition of the JAK and PI3K/Akt pathways may therefore lead to attenuation of these effects. It is an unanswered question if use of this drug combination will reduce the risk of blastic transformation.

A further important clinical question for taking forward such drug combinations, which these papers suggest are of major interest, is that we must define either appropriate clinical response criteria or a surrogate marker, such as reduction of *BCR/ABL* transcripts in CML. Reduction in splenomegaly is a key primary end-point in the phase III trials of JAK inhibitors that did not initially appear to be linked to survival or significant clinical benefit as assessed by accepted disease response criteria. Indeed, current response criteria 35–36 are complex and need to be refined to facilitate meaningful thorough assessment of clinical benefit as we use new therapies either alone or probable novel combinations identified from experiments, such as those described here.
